# Study of Oligonucleotides Access and Distribution in Human Peripheral Blood Mononuclear Cells

**DOI:** 10.3390/ijms23105839

**Published:** 2022-05-23

**Authors:** Manuel Fernández-Delgado, Luis Sendra, María José Herrero, Gladys G. Olivera-Pasquini, Alexander Batista-Duharte, Salvador F. Aliño

**Affiliations:** 1 Service of Hematology and Hemotherapy, Hospital General Universitario de Castellón, 12004 Castelló de la Plana, Spain; mfdezdm@gmail.com; 2 Farmacogenetics and Gene Therapy Group, Instituto de Investigación Sanitaria La Fe, Av. Fernando Abril Martorell, 106, 46026 Valencia, Spain; maria.jose.herrero@uv.es (M.J.H.); gladysmjiv@hotmail.com (G.G.O.-P.); alino@uv.es (S.F.A.); 3 Gene Therapy and Pharmacogenomics Group, Department of Pharmacology, Faculty of Medicine, University of Valencia, Av. Blasco Ibáñez 15, 46010 Valencia, Spain; 4 GC01 Immunology and Allergy Group, Maimonides Biomedical Research Institute of Cordoba (IMIBIC), Av. Menéndez Pidal, s/n, 14004 Córdoba, Spain; batistaduhartea@gmail.com; 5 Laboratório de Imunología Clínica, Dpto Analises Clinicas, Faculdade de Ciências Farmacêuticas, Universidade Estadual Paulista (UNESP), Rod. Araraquara-Jaú—Km 1, Campus Ville, 14800 Araraquara, Sao Paulo, Brazil

**Keywords:** flow cytometry, oligonucleotides, Treg, cell uptake, quenching, fluorescent labeling, PBMC, FOXP3, CD8+ Treg

## Abstract

Therapeutic oligonucleotides have achieved great clinical interest since their approval as drug agents by regulatory agencies but their access and distribution in blood cells are not completely known. We evaluated by flow cytometry the ability of short fluorescent scramble oligonucleotides (ON*) to access human peripheral blood mononuclear cells (PBMC) after incubating with ON* during 1 h and 7 days of culture follow-up ‘in vitro’. Blood samples were treated with chemically modified oligonucleotides (phosphorothioate backbone and 2′ O-Me ends) to resist nuclease digestion under culture conditions. The ON* internalization was determined after discarding the membrane-associated fluorescence by trypan blue quenching. Whereas the oligonucleotide accessed neutrophils and monocytes rapidly, achieving their maximum in 1 h and 24 h, respectively, lymphocytes required 7 days to achieve the maximum (80% of cells) transfection. The ON*ability to access lymphocyte types (T, B, and NK) and T cell subtypes (CD4+, CD8+, and CD4-CD8-) were similar, with T cells being more accessible. Regulatory CD4+ and CD8+ T cells were classified in low and high Foxp3 expressers, whose expression proved not to alter the ON* internalization during the first hour, achieving 53% of CD4+Foxp3+ and 40% of CD8+Foxp3+ cells. Our results contribute to understanding and improving the management of therapeutic ONs.

## 1. Introduction

The field of therapeutic oligonucleotides (ON) includes double-stranded small-interfering RNA, CRISPR-Cas9 single-guide RNAs, self-replicating messenger RNA (mRNA), and single-stranded antisense oligonucleotides (ASOs). Among these, ASOs are considered one of the most advanced oligonucleotide-based therapeutic modalities for personalized medicine, as evidenced by them having the highest number of New Drug Application approvals [1,2]. So far, ten single-stranded ASOs and four double-stranded small interfering RNAs (siRNAs) have received regulatory approval [3,4]. Although the greatest advances in the clinical application of ASOs have been largely focused on the treatment of certain rare genetic diseases, there is new research that pursues the extension of their clinical use in other important areas, including oncology, infectious and metabolic diseases, and vaccines, amongst others [5,6,7].

ASOs are short (usually 13–25 mer ONs) synthetic single-stranded nucleotides of DNA or RNA whose sequences are complementary to a target mRNA. They are designed to silence specific genes by interfering with the mRNA translation process, thus blocking protein synthesis [8]. ASO-DNA binding to target mRNA induces the activation of RNase H endonuclease, which catalyzes the cleavage of RNA in the RNA/DNA heteroduplex, and sterically blocks mRNA preventing its translation (both DNA and RNA ASOs). These two effects result in gene expression silencing. Other ASO-mediated silencing mechanisms include inhibition of 5′ cap formation, alteration of splicing process (splice switching), and steric hindrance of ribosomal activity. 

Despite the substantial progress in chemical modifications and understanding of the molecular mechanisms of action, pharmacokinetics, cell uptake, and subcellular distribution of oligonucleotides (ONs), there is still a great deal to learn [9]. Aspects such as delivery, off-target effect, and safety, still need to be resolved. In this study, we evaluated the cell access and distribution of short scramble fluorescent (Cy5 or FITC) labeled oligonucleotides (ON*) in blood lymphocytes, neutrophils, and monocytes. To protect the oligonucleotides from nuclease degradation and prolong their stability in blood, we employed a phosphorothioate-modified oligonucleotide backbone with 2′ O-Me modification in the three terminal nucleotides of the 5′ and 3′ ends. We evaluated the ON uptake by different lymphocyte types (T, B, and NK) and T cell subtypes (CD4+, CD8+, and CD4-CD8-), including regulatory T cells (CD4+CD25+FOXP3+ and CD8+FOXP3+). Finally, additional experiments were made to evaluate the effect of an oligonucleotide with the same sequence as ON* but unlabeled on the regulatory T cells expression of Foxp3 along time under the established experimental conditions. These results will be useful to understand the dynamics of ONs capture during their transit through the bloodstream and to optimize the study conditions when it is required to use long-term cultures of peripheral blood mononuclear cells (PBMCs) as a model to evaluate the immune response [10].

## 2. Results

### 2.1. Dose Selection of Different Sized Cy5-Oligonucleotide (ON*) Uptake by Leukocytes from Human Whole Blood

**Figure 1:**
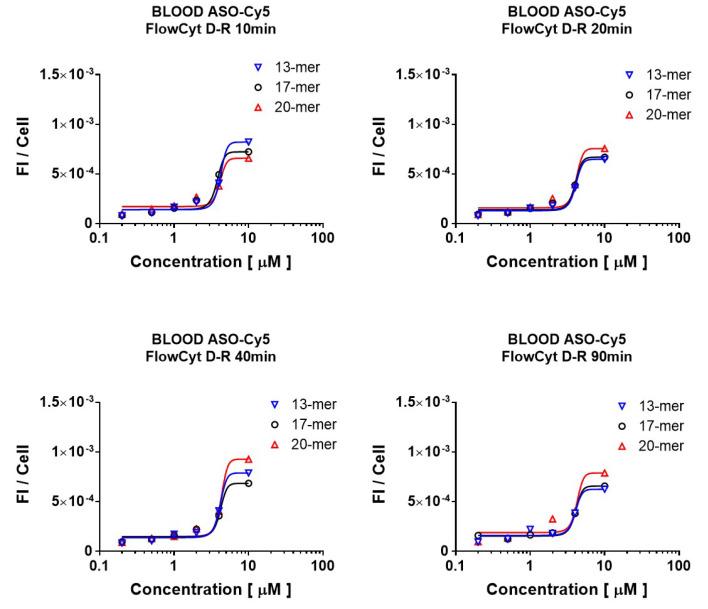
Dose-response fluorescent Cy5-ON (ON*) and size. Kinetics of Cy5 labeled oligonucleotide (ON*) on PBMC. Labeled 13-, 17- or 20-mer ONs were incubated at concentrations of 0.2, 0.5, 1, 2, 4 and 10 µM at 37 °C during 10, 20, 40, and 90 min. At each time point, the leukocytes (CD45+) were analyzed by flow cytometry to measure the ON* uptake and the emitted fluorescence, expressed as fluorescence intensity per cell (FI/cell). Lines through experimental points fit a sigmoidal dose-response model and were drawn with Graphpad Prism software.

The cell uptake of fluorescent oligonucleotides (ON*) with three different sizes (13-, 17-, and 20-mer) were administered at distinct concentrations (0.2, 0.5, 1, 2, 4, and 10 µM) was evaluated along time during 90 min. As shown in Figure 1, a similar pattern was observed in the four incubation times (10, 20, 40, and 90 min) evaluated. ON* concentrations lower than 1 µM mediated undetectable ON* uptake by leukocytes. However, thereafter, there was a sustained increase in ON* uptake in a similar manner with all three sizes. At 10 µM, size-depending differential uptake of ON* was observed. Higher concentrations were not tested since they provoke cell toxicity. Based on this study to select the optimal ON size and concentration, we decided to continue evaluating the ON* uptake kinetics in blood leukocytes employing a 4 µM concentration of a 20-mer ON*. This size is frequently used in gene silencing studies, mainly due to the favorable balance between cell accessibility and low off-target probability.

### 2.2. Culture Medium Selection Based on Leukocyte Viability along Time 

**Figure 2:**
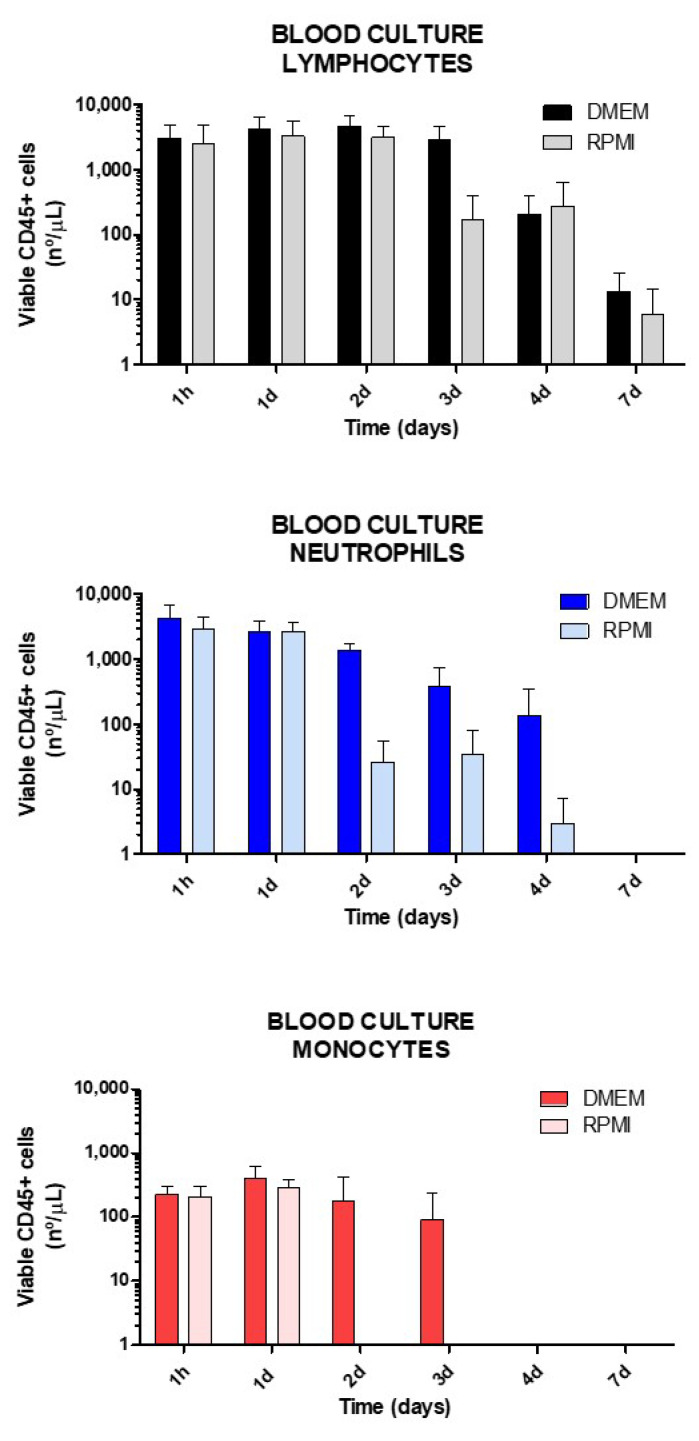
Comparison between RPMI and DMEM culture media in leukocyte viability for 7 days. 2.5 mL of blood samples were centrifuged and plasma was substituted with either DMEM or RPMI containing 25% of its own plasma and antibiotics. The viability of neutrophils, monocytes, and lymphocytes was determined by the 7AAD test in flow cytometry.

After the blood treatment conditions with ON* were defined, the viability of the different cell subtypes in DMEM and RPMI (containing 25% of own plasma) was evaluated over time (0, 15 min, 30 min, 45 min, 60 min, 1 day, 2 days, 3 days, 4 days, 7 days) in order to determine which culture medium offers advantages for neutrophils, lymphocytes, and monocytes survival. As can be observed in Figure 2, greater cell viability was achieved in DMEM culture. Whereas lymphocytes showed a longer survival rate (up to 7 days) with a similar viability rate with either DMEM or RPMI mediums, neutrophils and monocytes presented shorter viability (up to 4 and 3 days, respectively), especially with RPMI that offered shorter cell viability, particularly for monocytes (only 1 day).

### 2.3. Leukocyte Nuclear Access of Fluorescent FITC-ON 

**Figure 3:**
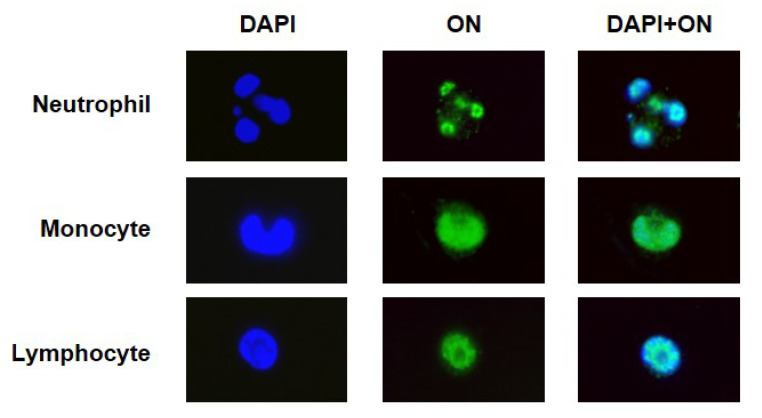
Location of fluorescent ON* in peripheral blood leukocytes treated with FITC-labeled ON* and incubated for 0.5 h at 0.5 µM. Then, the oligonucleotide was removed and PBMC in phosphate-buffered saline (PBS) solution was extended on a slide and dried. Cells were marked with DAPI and analyzed using a fluorescence microscope to observe the uptake of ON* by neutrophils, monocytes, and lymphocytes.

In order to study the ON*distribution inside different cell types, intracellular fluorescence was observed by fluorescence microscopy (Figure 3). ON* was dyed with green FITC fluorophore and this fluorescence could be observed co-localized with DAPI inside the nucleus of lymphocytes, neutrophils, and monocytes, suggesting that ON can access and concentrate within the nuclear compartment.

### 2.4. Kinetics of FITC-ON Internalization in Leukocytes

**Figure 4:**
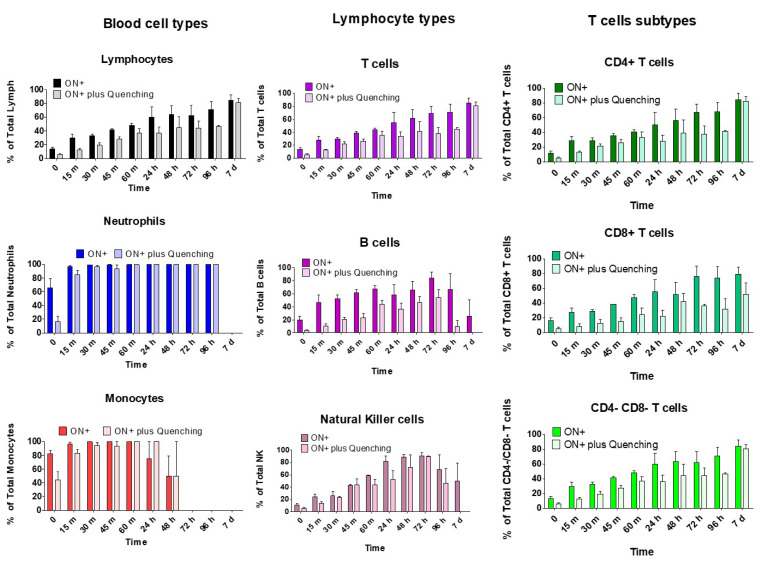
Fluorescent leukocytes (%) after 20-mer ON* treatment at 4 µM with DMEM incubation before and after Quenching. The bar graphics represent the percentage of viable cells positive for green fluorescence provided by ON*. Percentage of viable cells with fluorescence before and after TB membrane fluorescence quenching were compared. Different leukocyte cells (lymphocytes, neutrophils, and monocytes), lymphocyte types (T cells, B cells, and NK cells), and T cells subtypes (CD4+, CD8+, and CD4-CD8-) are depicted.

To monitor the internalization of FITC-labeled ON in different leukocyte populations, we exploited the ability of the non-penetrating trypan blue (TB) dye to quench the green fluorescence of the FITC-labeled ONs attached to the cell surface to detect only that of the internalized compound [11,12,13,14]. In an attempt to evaluate the ON* distribution among the different subpopulations of lymphocytes, we evaluated CD3+ cells (T cells), CD19+ cells (B cells), and CD56+ cells (NK cells), and CD4+, CD8+, and CD4-CD8- T cells subpopulations. As seen in Figure 4, we differentiate two populations: ON+ (fluorescent cells, without considering dye location) and ON+ plus quenching (fluorescent cells even after TB quenching, indicating an intracellular location of ON*, since membrane fluorescence is quenched). It is shown that most of the ON* was internalized and remained detectable inside the cells throughout the period of study. Interestingly, it was observed that most of the viable lymphocytes were ON+ after 7 days. CD4+ T lymphocytes proved the highest survival rate at 7 days of incubation (above 70% on average) in the presence of ON*, whose internalization along time greatly differed among cell populations. 

The most rapid uptake of ON* occurred in neutrophils, in which, 100% of cells had internalized the ON* after 30 min of incubation. Monocytes also uptook the ON* very quickly and only required 1 h to achieve 100% of cell positive for ON* after quenching. On the contrary, lymphocytes showed slower kinetics of ON* internalization, not achieving its maximum (90% of cells) until day 7, and only 50% of cells had internalized the ON* on day 4. This behavior was also observed in T cells, with similar results in CD4+ and CD4-CD8- T cells subtypes, whereas the internalization of ON* in CD8+ did not reach more than 80% of cells. 

On the other side, B and NK cells achieved their maximum internalization (60% and nearly 100%, respectively) on day 3, and afterward, the proportion of fluorescent cells after quenching decayed, maybe due to the loss of viability. 

The amount (cells/µL) of ON*-, ON*+, and ON*+ plus quenching cells of each subtype after treatment with 20-mer ON* at 4 µM concentration and 7 days DMEM incubation, are shown in Table 1. 

### 2.5. Fluorescence Intensity of FITC-ON in Leukocytes

**Figure 5:**
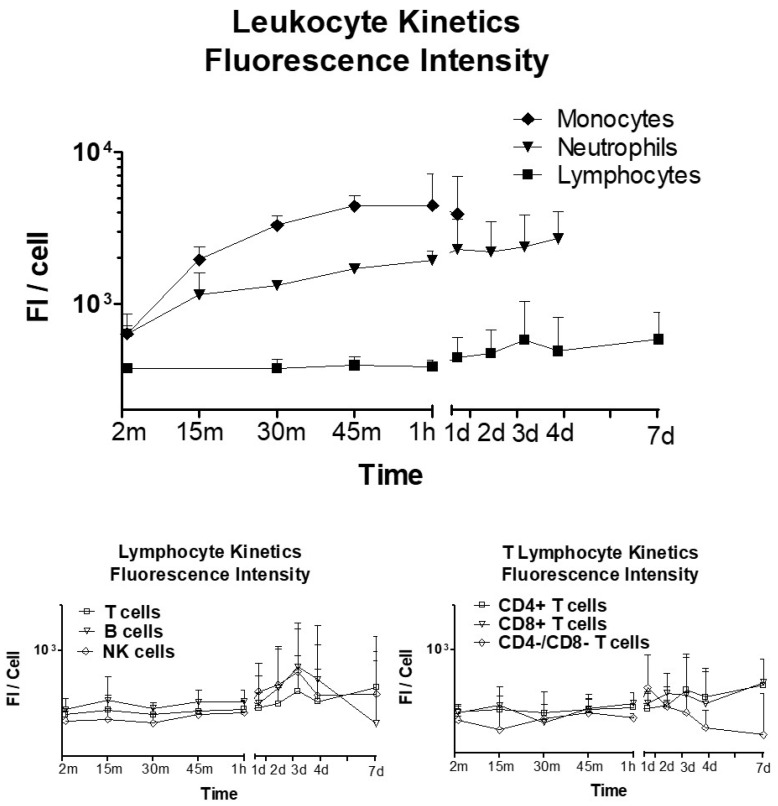
Fluorescence intensity per cell in blood cells treated with fluorescent 20-mer ON leukocytes at 4 µM and DMEM incubation + Quenching. Monocytes vs neutrophils *p* < 0.05; monocytes vs lymphocytes *p* < 0.001; neutrophils vs lymphocytes *p* < 0.01.

The fluorescence intensity per cell of the internalized ON* (fluorescence after quenching) was also analyzed. Figure 5 shows different kinetics of ON* access to the cells depending on their type. Cells were incubated for 2, 15, 30, 45, and 60 min, and then, the ON* was removed and cells were maintained for 7 days in culture with DMEM plus plasma. It was observed that ON* uptake occurred very rapidly in monocytes, achieving a maximum fluorescence plateau after one hour of incubation. Neutrophils showed a gradual increase of ON* fluorescence along the whole period of the study, being faster during the first hour and slower onwards until day 4. Lymphocytes exhibited lower and slower uptake of fluorescent ON* compared to monocytes (*p* < 0.001) and neutrophils (*p* < 0.01). Their fluorescence began to rise on the first day of cell culture and increased slightly until the end of the experiment on day 7 (with a peak of fluorescence on day 3, also observed with T, B, and NK cells). CD4+ and CD8+ T cells showed greater intracellular fluorescence than CD4-CD8- T cells on the last days. Lymphocyte types and T cell subtypes did not show statistically significant differences among them. 

### 2.6. Access of FITC-ON to Regulatory T Cells

**Figure 6:**
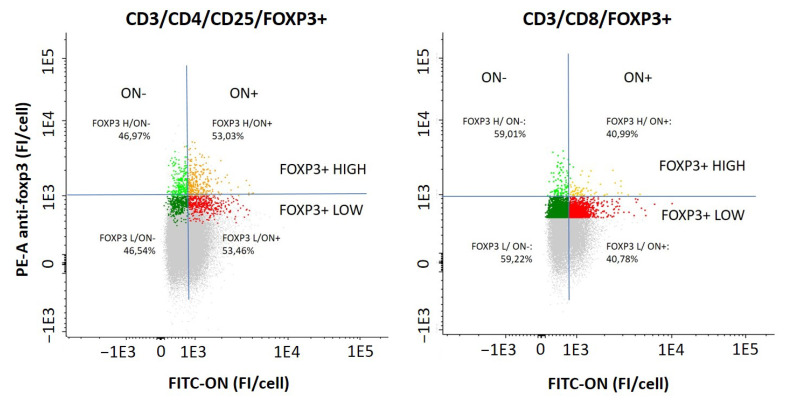
The panel shows representative cytometry plot of fluorescence intensity of ON:FITC vs Foxp3:PE of CD3+CD4+CD25+Foxp3+ and CD3+CD8+Foxp3+ cells population. Cells were divided into ON-/ON+ depending on their FITC fluorescence (ON- < 1E3; ON+ > 1E3) and classified them upon their Foxp3 expression (low: fluorescence < 1E3; high: fluorescence > 1E3).

To confirm the access of the ON* into regulatory T (CD4+ and CD8+) cells, we evaluated the proportion of CD4+CD25+Foxp3+ and CD8+Foxp3+ T cells that presented FITC fluorescence after incubating whole blood cells with ON* at 4 µM concentration for 1 h (Figure 6) by flow cytometry. ON- vs ON+ populations of both CD4+ and CD8+ T cells were differentiated (left and right of the vertical blue line, respectively) and, depending on the level of Foxp3 protein expression, we also identified low and high expression subpopulations (below and above the horizontal blue line, respectively). It can be observed that ON* could access both CD4+ and CD8+ regulatory T cells, with a slightly higher ability to enter CD4+CD25+Foxp3+ cells (53% vs 40%). We observed that the Foxp3 level of expression did not alter the ON* access efficacy either in CD4 or CD8 regulatory T cells populations, showing both low and high Foxp3 expressers the same level of ON internalization. 

**Figure 7:**
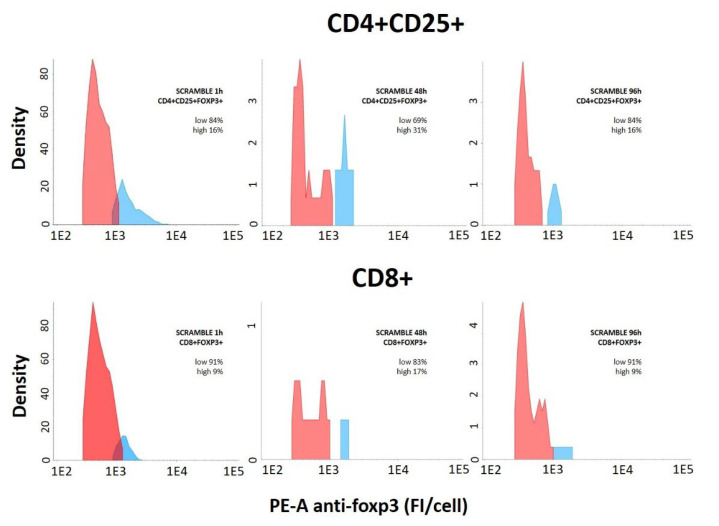
The figure shows representative plot of cell distribution according to their level of Foxp3 expression. CD3+CD4+CD25+Foxp3+ (upper panel) and CD3+CD8+Foxp3+ (lower panel) cells are represented. In each case, two different populations can be clearly differentiated upon their Foxp3 expression (low: fluorescence < 1E3; high: fluorescence > 1E3).

The distribution of regulatory T cells according to their Foxp3 expression level along time (Figure 7) proved the existence of two differentiated populations (low Foxp3 expressers, in red; and high Foxp3 expressers, in blue), confirming the threshold employed to differentiate these subpopulations within Figure 6. It was observed that the high expresser population increased on day 2 up to 30% of cells and it decreased hereon, being 0% on day 7. The complete data of this analysis are presented in Table 2.

## 3. Discussion

In the present study, FITC-conjugated oligonucleotides (ON*) uptake by PBMC and their internalization during 7-day culture in DMEM or RPMI containing 25% of own plasma has been evaluated by flow cytometry. With this analysis, we aimed to shed some light on the process occurring immediately after the intravenous administration of ONs and elucidate its pattern of distribution among the blood cells. The ON* was incubated for 1 h in complete blood and then removed, and cells were cultured for an additional period of 7 days in a culture medium and own plasma. DMEM medium offered advantages in the survival study, permitting lymphocytes to survive for at least 7 days, whereas monocytes and neutrophils died after 3 and 4 days of culture, respectively. In these conditions, cells were analyzed in the presence and absence of trypan blue, which suppresses the fluorescence associated with ON* molecules attached to the cell membrane by quenching. Results show that ON* accessed the cells very rapidly in neutrophils and monocytes, achieving 100% of cells before 1 h of incubation. However, lymphocytes showed slower internalization of the ON*, requiring 7 days to achieve 80% of positive cells. The kinetics of fluorescence intensity was also different among the different cell types. In addition, flow cytometry permitted us to identify the regulatory CD4+ and CD8+ T cells as low and high expressers of Foxp3, and their distribution along time and we observed that our ON* could also access these regulatory T cells. 

The ONs employed in the present work harbor chemical modifications that guarantee plasma stability and permit the maintenance of their RNAse H activity when the ON interacts with its target. These chemical modifications consist of both phosphorothioated (PS) bonds (where sulfur substitutes for one non-bridging phosphate oxygen) within the ON backbone, and 2′ O-Me modification within the first 3 nucleotides of both upstream and downstream of the ON sequence. Besides, PS-modified ONs offer the advantage that can also access the cells without the use of transfection or electroporation [15]. Cellular uptake and trafficking of ONs depend on their structure (mainly length of the oligonucleotide and chemical modifications), concentration, and exposure conditions, and can vary among cell types [16]. The length of the ON determines its target selectivity and affects its ability to access cells. The optimal length as an antisense molecule has been established in 20-mer since it is believed that offers the best risk/benefit ratio, considering that can access most of the cells and permits specifically identifying its target with a very low risk of off-target events. ‘In vitro’ studies using ONs are often performed in established cell line cultures or in purified cells [17,18]. We decided to use human complete blood to study their behavior in the milieu where they are administered. At the optimal concentration of 4 µM, the ONs accessed the cells efficiently and we did not observe differences in their uptake due to their sizes (13, 17, and 20-mer). For this reason, all the other experiments were performed with 20-mer ON since could offer a better safety profile without apparent limitations in target accessibility. The ability of ONs to access the cells of interest and their subsequent cell location determine their biological activity. Different membrane-associated proteins that bind ONs and mediate their cellular uptake have been identified, including cell surface receptors, such as integrins (e.g., Mac-1/CD18), G-protein-coupled receptors, Stabilin, a scavenger receptor and epidermal growth factor receptor (EGFR) [14,16]. Although it has been observed that 35-mer, or larger, single-stranded ON inhibits clathrin-mediated endocytosis or blocks the entry receptor nucleolin [19,20] regardless of their sequence, the mechanisms that mediate intracellular uptake and productive target engagement by ONs are not fully understood yet. Therefore, determining the extent and mechanism of ONs uptake by cells, as well as their subcellular distribution, is important for evaluating their therapeutic potential [13]. 

In our study, extended survival of blood cells was required and, since there is evidence that culture medium can influence cell proliferation and cell viability [21], we decided to evaluate which of the two most used culture media, RPMI or DMEM, was more appropriate to culture leukocytes from peripheral blood. Although both media, widely used for cell cultures of the immune system, did not significantly alter the survival of lymphocytes, DMEM mediated higher neutrophils and monocytes viability rate in our study, supporting the results reported by other authors [21,22,23,24]. We detected that lymphocytes showed the highest survival rate, reaching day 7 with approximately 10 viable cells per µL. Neither neutrophils nor monocytes survived on day 7, but they exhibited a higher rate of survival in DMEM throughout their four days of survival. On day 4 of culture, more than 100 viable neutrophils/µL could be detected with DMEM whereas less than 10 could be quantified with RPMI. Regarding monocytes, DMEM prolonged their survival until day 3 while all cells died on day 1 with RPMI medium. 

The presence of ONs inside the cells and their persistence for 7 days of culture under the previously established conditions was determined, incubating the cells with 4 µM 20-mer ON* for 1 h and then removing it and culturing cells in DMEM+P. We studied the ON fluorescence within the three main blood cell types under fluorescence microscopy. We could observe that ON accessed lymphocytes, neutrophils, and monocytes since the FITC fluorescence of ON* was localized both in membrane/cytoplasm and within the nucleus, as demonstrated by its co-localization with nuclear DAPI staining. The number of cells that internalized the ON* was also determined by flow cytometry. To eliminate the fluorescence emitted by the ON* attached to the cell surface, we quenched it with trypan blue, which absorbs green-yellow light with a maximum absorbance wavelength of around 590 nm emitted by fluorescent molecules thus quenching FITC in flow cytometry [25,26]. This permitted differentiating the cells that had internalized the fluorescent oligonucleotide from those that had it attached to the cell membrane. Whereas in neutrophils and monocytes, practically 100% of the cells internalized ON* very early (in less than 1 h), only 40% of lymphocytes resulted positive for ON* fluorescence, which achieved their maximum ON* internalization (near 80%) on day 7. This limitation in ON* access to lymphocytes was also observed when evaluating the kinetics of fluorescence intensity in the different cell types. Clear differences were observed when compared to monocytes, which reached their fluorescence peak during the first hour (11-fold higher than lymphocytes), and neutrophils, whose fluorescence rapidly increased during the first hour (5-fold higher than lymphocytes), and the peak was reached on day 4 after stabilizing since day 1. Lymphocytes exhibited lower and slower uptake of fluorescent ON*. Their fluorescence did not rise until day 1 of culture and then increased slightly until day 7 (with a peak of fluorescence on day 3, as also observed with T, B, and NK cells). CD4+ and CD8+ T cells showed greater intracellular fluorescence than CD4-CD8- T cells on the last days. Although it has been suggested that serum nucleases could detach the fluorescent dye from unmodified oligonucleotides and facilitate its free access to cells [27], this effect must be very limited in our experiments since the chemically modified ONs employed present high nuclease resistance. 

After observing the possibility of studying the kinetics of ON access to lymphocytes in long periods of culture and the ability of these molecules to be internalized and reach the nuclei of immune cells, we evaluated the ON internalization in the reduced population of regulatory T cells. We observed that ON* could also access both CD4+CD25+Foxp3+ and CD8+Foxp3+ regulatory T cells (53% and 40%, respectively). Furthermore, the Foxp3 expression analysis permitted differentiation into two subtypes of these cells (low and high expressers), which showed no difference in oligonucleotide access rate. When the regulatory T cells were plotted in a graph distributing the population, according to their Foxp3 level of expression (anti-Foxp3 antibody fluorescence), the low and high expresser subpopulations were clearly defined and their evolution along time could be evaluated. Thus, approximately 15% of CD4+CD25+Foxp3+ cells were high Foxp3 expressers 1 h after treatment with scramble ON. This ratio increased to 30% on day 2, decreased to basal levels on day 4, and completely disappeared on day 7 of culture. In CD8+Foxp3+ cells, we observed the same behavior, high expressers’ proportion being always slightly lower, though. These results arouse the interest in developing strategies based on antisense oligonucleotides targeting FOXP3 with the aim of modulating its expression to minimize immune tolerance responses. This could present great translational application of oligonucleotides in immune activation treatments, such as vaccines and antitumor drugs. In this sense, whereas preventive antitumor vaccines have proved to be very efficacious, the therapeutic ones (administered post-tumor) have not. Recently, our group has reported [17] in a murine melanoma model that the antitumor efficacy of these vaccines can be partially recovered (approximately 50%) when the FOXP3 gene was silenced with antisense oligonucleotides before the vaccine administration. These data also suggest the relevance of knowing the Foxp3 expression level to support the clinical management in different scenarios with imbalanced immunity function, as well as helping to evaluate ‘in vitro’ pharmacological responses in immune cells using whole blood or unfractionated PBMCs as a model [10].

Finally, we observed a general pattern, but especially in lymphocytes, that exist a proportion of cells refractory to ON access could be due to the co-existence of naïve and differentiated T cells in the blood. Cell differentiation is a dynamic process that has been more minutely studied in exhaust T cells [28,29], whose phenotype is not fully defined. In this sense, the progression to terminal stages must be correlated with the cell differentiation phases, where the processes related to response capacity and exchange with the extracellular microenvironment must play an important role. We suggest that naïve T cells could be associated with facilitated access to ON but not the differentiated T cells. In our case, most surviving cells are ON positive and this could indicate that elder terminal cells, with limited ON access, die earlier than naïve lymphocytes.

## 4. Materials and Methods

### 4.1. Antibodies

All antibodies were purchased at Becton-Dickinson Inmunocytometry Systems, San Jose, CA 95131- USA. We employed the following: CD45 V500-C, REF: 653806; CD19 (SJ25C1) PeCy7, REF: 341113; CD20 (L27) V450, REF: 655872; CD56 (MY31) PE, REF: 34109; CD3 (SK7) APC, REF: 345767; CD4 (RPA-T4) V450, REF: 560345; CD8 (SK1) FITC, REF: 345772; CD25 (M-A251) APC. REF: 555434; FoxP3 (259D/C7) PE. REF: 560046. Other products required for cell staining were: BD Via-Probe, REF: 555816; VersaLyse. Lising solution, REF: A09777; Buffer Human FoxP3 Buffer A (10x), REF: 51-9005450; Buffer Human FoxP3uffer B (50x), REF: 51-9005450; Fixable Viability Stain 510 (FVS510). BV-510, REF: 564406; Lysing Buffer, REF: 555899; PharmingenStain Buffer (BSA), REF: 554657. BD TRUCOUNT TUBES (REF: 342447, Becton-Dickinson) was used for cell counting.

### 4.2. Oligonucleotides (ONs)

All the oligonucleotides were purchased from Integrated DNA Technologies (Coralville, IA USA) and modified as 2′-OMe-PS-ASO to increase their resistance to exo- and endonucleases. For the first ON dose-response studies, we employed 3 different ONs (13-, 17- and 20-mer) dyed with Cy5 with the following sequences: 13-mer, 5′-TCGAACGGACTCT-3′; 17-mer, 5′-TCGAATCCGAGGACTCT-3′; 20-mer, 5′-TCGAATTGACCGAGGACTCT-3′. The long-term study of ON access into cells was carried out with a FITC dyed 20-mer scrambled ON with the following sequence: 5′-TCGAATTGACCGAGGACTCT-3′. To study the Foxp3 expression activity, ON sequences were the same as FITC labeled scramble ON but without dye: scramble ON: 5′-TCGAATTGACCGAGGACTCT-3′ (20 bp). 

### 4.3. ON Dose-Response Study in Human Whole Blood

Three oligonucleotides (ONs) of 13-, 17- and 20-mer length 2′-OMe-PS-ASOs, labeled with cyanine-5 (Cy-5) were employed to determine the optimal ON size and concentration mediating the highest fluorescence without toxicity. Labeled ONs of either 13-, 17- or 20-mer were incubated independently at concentrations of 0.2, 0.5, 1, 2, 4, and 10 µM at 37 °C for 10, 20, 40, and 90 min in human whole blood (n: 6). At each time and for each cell concentration, 50 µL cell suspension was taken, properly dissolved in phosphate buffer saline (PBS pH 7.4), and analyzed by means of flow cytometry to measure the relative fluorescence (as FI/cell) in CD45+ cells. A Gallios cytometer (Beckman Coulter, Brea, CA, USA) was used in these analyses.

### 4.4. Comparative Study of Culture Medium Survival 

Blood samples were collected from healthy donors for 2 h prior to beginning the protocol in heparin-lithium tubes. 2.5 mL blood was centrifuged at 1500× *g* for 10 min. 750 µL of plasma was collected and blood cells were resuspended with 2.5 mL of DMEM (n: 3; Dulbecco’s Modified Eagle Medium, Lonza, Morristown, NJ, USA) or RPMI (n: 3; Roswell Park Memorial Institute Medium, Gibco, Thermo Fisher Scientific, Waltham, MA, USA) medium containing 25% of own plasma and 1% penicillin-streptomycin mix (Lonza, NJ, USA). The first analysis of cell viability was performed on a 200 µL sample at time 0 by flow cytometry. The remaining blood with DMEM or RPMI was maintained in culture at 37 °C and 5% CO2 for 7 days. Samples were collected at 1 h, 1 day, 2 days, 3 days, 4 days, and 7 days and washed twice, then the viability of CD45+ (leukocytes) cells was determined by flow cytometry employing the 7 AAD test. Cell types were identified by size and morphology with Side and Forward Scattering analyses in a BD FACS Canto II Flow Cytometer (Becton-Dickinson Immunocytometry Systems, CA, USA) and BD FACS Diva software (Becton Dickinson, CA, USA) as follows: Collect 100 µL of the sample, add monoclonal antibodies, vortex briefly and incubate for 20 min in darkness. Then, 1 mL of Versalyse lysing buffer was added, vortexed immediately for 1 s, and incubated for 10 min at room temperature in darkness. The sample was washed with PBS twice, resuspended with 500 µL of PBS, and 100 µL was added to a Tru Count tube, and analyzed.

### 4.5. Intracellular Location of FITC-ON Fluorescence by Fluorescence Microscopy

FITC-dyed 20-mer ON was added to two independent 3 mL blood samples from a 0.5 µM final concentration. It was incubated for 30 min at 37 °C. Then, it was centrifuged at 1500× *g* for 10 min, the plasma was removed and we collected the leucocyte white layer, which was added to a microscope slide and extended. After it dried, a drop of DAPI solution (1 mg/mL, Thermo Fisher Scientific, MA, USA) was added and it was observed under a Nikon Eclipse 50i fluorescence microscope (Nikon Instruments, Melville, NY, USA). 

### 4.6. ON Uptake Kinetics in Human Whole Blood

During the two hours prior to beginning the experiment, 5 mL blood samples from healthy donors (n: 4) were collected in heparin-lithium tubes. Each sample was centrifuged at 1500× *g* for 5 min and plasma was separated from cells and preserved. Cells pellet was resuspended with 1 mL DMEM with 25% human plasma (DMEM+P) obtained after blood centrifugation. This blood with DMEM was treated with the 20-mer FITC-fluorescent oligonucleotide at a 4 µM final concentration. The mix was incubated at 37 °C and 20 µL and samples were collected at different time points (0′, 15′, 30, 45′ and 1 h), diluted in 280 µL DMEM solution containing 7AAD and the pool of antibodies required for cell subtype differentiation. Samples were incubated for 5 min in darkness, and red cells were lysed and washed. Cells were resuspended in PBS, added to a Tru Count tube, and analyzed by flow cytometry. After 1 h, the remaining sample was washed twice with DMEM to remove the oligonucleotide. The cell pellet was resuspended with DMEM + human plasma (25%) and antibiotics (penicillin-streptomycin, 1%) and incubated at 37 °C and 5% CO_2_ to repeat the antibody-staining and flow cytometry in 20 µL samples collected 1, 2, 3, 4 and 7 days after the treatment with the oligonucleotide, as previously described. To discard the ON fluorescence that could be present within its membrane outside the cell, it was quenched with Trypan blue 0.4% (Thermo Fisher Scientific, MA, USA). Trypan blue 0.4% was added to the sample to a final 1:3 ratio (TB:sample) to quench the extracellular green fluorescence and be able to identify the FITC-positive cells that had internalized the ON*. Their intracellular fluorescence intensity per cell (n: 4) was also quantified by flow cytometry as above described. 

### 4.7. Determination of ON* Access to CD4 and CD8 T Cells According to Their Foxp3 Expression

CD4 and CD8 regulatory T cells expression of Foxp3 and the access of FITC-ON to these cells were analyzed by flow cytometry. Samples (n: 2) were treated with scramble FITC-oligonucleotide at 4 µM as above described. These samples were incubated for 1 h at 37 °C and 5% CO_2_. Then, a 20 µL sample was collected and diluted with 280 µL DMEM. Red cells were lysed and the mixture was washed twice. Then cells were stained with FVS dye to determine their viability and washed twice again. After that, cells were stained with CD3, CD4, CD8, and CD25 antibodies and incubated for 20 min at room temperature in darkness. Since foxp3 is expressed intracellularly, cells had to be fixed and permeabilized. Thus, after washing, the cells were resuspended in wash buffer and specific fixative buffer (Human Foxp3 Buffer A) and incubated for 10 min at room temperature in the dark. Then, after a new wash step, cells were permeabilized with buffer and incubated for 30 min at RT in darkness. After new washes, the anti-Foxp3 antibody was added at recommended concentration and incubated for 30 min at RT in the dark. After a new wash step, cells were resuspended with buffer and added to Tru Count Tube to be analyzed by flow cytometry to observe the distribution of cells population regarding their expression of Foxp3 and its effect on ON* access to cells. Other 5 blood samples were treated with scramble not labeled ON for 1 h, then, were washed twice with DMEM and after that, the cell pellet was resuspended with DMEM + plasma (25%) + antibiotics (penicillin-streptomycin, 1%) and incubated under the same conditions. Samples were collected 2, 4, and 7 days after ON treatment and analyzed following the same protocol as 1 h sample to evaluate the population distribution according to their expression of Foxp3 and the evolution of this process along time.

### 4.8. Statistical Analysis

Cells transfection and fluorescence intensities of different types of PBMCs were compared by ONE-WAY ANOVA and Bonferroni post-test, performed on Graphpad Prism v.05 software. 

## 5. Conclusions

Our results contribute to increasing the knowledge about ON access to PBMCs aiming to optimize their use as therapeutic agents and modulate their application based on their cellular and molecular targets. The access of scramble oligonucleotides with similar characteristics to those employed in therapeutics to PBMCs show different kinetics among the types of leukocytes (monocytes = neutrophils > lymphocytes) and their maximum entry levels (% of ON+ cells after quenching) were achieved at markedly different time points: 1 h and 7 days, respectively. The fluorescence intensity per cell showed the same behavior during the study period, being the maximum achieved in neutrophils (4d) and monocytes (1d) up to 5- and 11-fold higher than the observed in lymphocytes (7d), respectively. Although ON* could access most of PBMCs, there was a subpopulation (20%) of lymphocytes that appeared as refractory to their entry during the entire period of study. The population of regulatory CD4+ and CD8+ T cells presented an equivalent rate of ON* uptake and could be classified by flow cytometry as low and high Foxp3 expressers. The evaluation of this nuclear factor expression could have diagnostic value and serve to monitor the therapeutic target.

## Figures and Tables

**Table 1:** Absolute values (cells/µL) of leukocytes, lymphocyte types, and T cells subtypes after treatment with fluorescent 20-mer ON* at 4 µM and DMEM incubation at each evaluated time. Three different groups (ON-, ON+, and ON+ after quenching) are shown. Avg.: average; m: minutes; h: hours; d: days.

ON-	Lymphocytes		Neutrophiles		Monocytes		ON-	T cells		B cells		NK cells		ON-	CD4+ T cells		CD8+ T cells		CD4-CD8- T cells	
Time	Avg.	SD	Avg.	SD	Avg.	SD	Time	Avg.	SD	Avg.	SD	Avg.	SD	Time	Avg.	SD	Avg.	SD	Avg.	SD
0	**75.93**	40.02	**51.58**	57.50	**1.89**	0.94	0	**51.53**	24.25	**10.33**	7.32	**13.67**	8.56	0	**36.07**	19.54	**11.93**	4.80	**3.51**	1.25
15 m	**105.18**	69.69	**7.16**	8.34	**1.06**	1.87	15 m	**73.34**	44.39	**13.32**	10.92	**18.60**	14.75	15 m	**49.00**	31.16	**20.97**	12.71	**3.36**	3.24
30 m	**80.50**	38.95	**2.44**	2.85	**0.14**	0.28	30 m	**58.90**	23.97	**8.41**	6.36	**13.37**	8.90	30 m	**39.95**	19.04	**15.47**	4.55	**3.45**	1.14
45 m	**66.18**	28.18	**1.24**	2.08	**0.00**	0.00	45 m	**49.05**	17.74	**6.38**	4.34	**10.94**	6.36	45 m	**33.14**	14.03	**13.08**	3.57	**2.89**	1.50
60 m	**39.37**	13.68	**0.41**	0.78	**0.05**	0.10	60 m	**37.46**	5.07	**3.83**	2.44	**6.10**	3.10	60 m	**26.25**	5.61	**9.51**	2.64	**1.72**	0.47
24 h	**23.38**	20.55	**0.04**	0.08	**0.00**	0.00	24 h	**18.46**	15.47	**3.16**	3.23	**1.83**	2.02	24 h	**13.10**	11.65	**4.65**	3.45	**0.57**	0.54
48 h	**26.70**	23.99	**0.00**	0.00	**0.00**	0.00	48 h	**22.50**	19.48	**2.99**	3.18	**2.26**	3.03	48 h	**15.42**	14.08	**6.07**	4.64	**0.60**	0.52
72 h	**14.07**	10.72	**0.00**	0.00	**0.00**	0.00	72 h	**13.23**	10.35	**0.45**	0.53	**0.84**	1.01	72 h	**10.63**	9.92	**2.19**	2.58	**0.47**	0.61
96 h	**18.30**	21.75	**0.00**	0.00	**0.00**	0.00	96 h	**16.46**	20.80	**0.22**	0.43	**0.43**	0.86	96 h	**11.95**	13.64	**5.73**	6.77	**0.43**	0.86
7 d	**3.38**	3.91	**0.00**	0.00	**0.00**	0.00	7 d	**3.23**	4.00	**0.00**	0.00	**0.00**	0.00	7 d	**2.18**	2.98	**0.93**	0.94	**0.13**	0.15
**ON+**	**Lymphocytes**		**Neutrophiles**		**Monocytes**		**ON+**	**T cells**		**B cells**		**NK cells**		**ON+**	**CD4+ T cells**		**CD8+ T cells**		**CD4-CD8- T cells**	
Time	**Avg.**	SD	**Avg.**	SD	**Avg.**	SD	Time	**Avg.**	SD	**Avg.**	SD	**Avg.**	SD	Time	**Avg.**	SD	**Avg.**	SD	**Avg.**	SD
0	**7.86**	7.13	**93.13**	62.77	**10.50**	6.50	0	**8.12**	4.43	**1.80**	1.08	**1.43**	0.80	0	**5.17**	3.23	**2.39**	1.24	**0.56**	0.39
15 m	**39.32**	23.47	**183.54**	75.35	**22.30**	13.98	15 m	**26.37**	14.38	**8.51**	7.01	**4.70**	2.85	15 m	**18.07**	10.96	**7.14**	4.07	**1.15**	0.96
30 m	**42.59**	24.28	**172.56**	73.61	**15.96**	9.97	30 m	**29.61**	17.16	**7.19**	3.97	**7.09**	4.88	30 m	**19.79**	12.50	**6.61**	3.18	**1.78**	0.41
45 m	**46.38**	19.16	**166.69**	35.81	**17.65**	8.51	45 m	**29.87**	11.35	**8.42**	3.52	**8.09**	4.64	45 m	**19.48**	10.37	**8.28**	2.18	**2.27**	0.55
60 m	**45.52**	14.17	**130.28**	20.84	**14.02**	5.74	60 m	**29.94**	8.11	**7.38**	2.75	**6.15**	4.88	60 m	**18.89**	7.12	**8.43**	1.67	**2.64**	1.05
24 h	**27.29**	20.03	**52.96**	22.72	**3.14**	2.23	24 h	**16.08**	8.72	**3.60**	3.65	**7.57**	7.87	24 h	**9.92**	7.49	**4.23**	1.81	**1.93**	0.70
48 h	**33.79**	29.94	**43.43**	46.27	**0.94**	1.25	48 h	**19.56**	16.94	**3.69**	3.69	**10.04**	11.18	48 h	**14.74**	13.13	**4.38**	2.69	**2.76**	2.28
72 h	**35.97**	15.66	**33.78**	38.40	**0.00**	0.00	72 h	**27.85**	12.55	**2.52**	2.10	**6.86**	3.31	72 h	**19.34**	10.43	**8.99**	7.12	**1.19**	1.52
96 h	**27.16**	18.81	**7.06**	2.12	**0.00**	0.00	96 h	**22.72**	14.60	**1.77**	1.54	**4.75**	5.43	96 h	**16.13**	11.30	**5.82**	3.01	**2.05**	1.33
7 d	**18.27**	7.07	**0.00**	0.00	**0.00**	0.00	7 d	**17.72**	6.16	**0.12**	0.24	**0.44**	0.71	7 d	**12.80**	5.33	**4.04**	2.17	**0.88**	0.73
**ON+/Q**	**Lymphocytes**		**Neutrophiles**		**Monocytes**		**ON+/Q**	**T cells**		**B cells**		**NK cells**		**ON+/Q**	**CD4+ T cells**		**CD8+ T cells**		**CD4-CD8- T cells**	
Time	**Avg.**	SD	**Avg.**	SD	**Avg.**	SD	Time	**Avg.**	SD	**Avg.**	SD	**Avg.**	SD	Time	**Avg.**	SD	**Avg.**	SD	**Avg**.	SD
0	**47.44**	26.79	**33.50**	23.11	**7.99**	6.55	0	**4.89**	3.06	**0.32**	0.26	**0.71**	0.46	0	**2.67**	1.74	**1.53**	1.35	**0.70**	0.78
15 m	**170.35**	98.82	**139.75**	79.88	**18.59**	14.90	15 m	**10.09**	4.39	**0.98**	0.72	**1.40**	0.98	15 m	**7.23**	5.40	**2.31**	0.83	**1.18**	1.06
30 m	**231.13**	168.53	**175.59**	110.83	**24.97**	30.07	30 m	**24.19**	23.75	**3.34**	4.17	**4.56**	3.68	30 m	**16.84**	19.94	**4.81**	3.95	**2.52**	1.08
45 m	**262.29**	166.86	**198.16**	108.45	**32.74**	40.20	45 m	**15.72**	15.94	**3.37**	3.01	**6.00**	4.55	45 m	**13.03**	8.50	**6.01**	4.66	**2.66**	1.82
60 m	**156.96**	38.35	**110.90**	24.16	**14.95**	9.42	60 m	**22.03**	8.08	**5.27**	7.22	**3.90**	2.35	60 m	**12.65**	6.09	**6.40**	2.16	**3.00**	0.98
24 h	**74.35**	25.85	**41.22**	10.62	**6.55**	3.67	24 h	**17.77**	13.65	**3.44**	4.48	**5.52**	6.15	24 h	**9.61**	8.90	**5.39**	3.40	**2.83**	1.64
48 h	**57.24**	8.07	**34.14**	0.16	**1.37**	1.94	48 h	**17.33**	5.80	**0.83**	0.47	**3.58**	0.96	48 h	**8.96**	3.69	**6.08**	1.22	**2.29**	0.88
72 h	**23.21**	4.30	**9.73**	1.51	**0.00**	0.00	72 h	**9.75**	4.96	**1.07**	0.74	**2.81**	1.39	72 h	**5.26**	3.67	**3.68**	1.69	**0.82**	0.40
96 h	**25.77**	13.44	**5.26**	2.76	**0.00**	0.00	96 h	**17.71**	8.98	**0.20**	0.35	**2.84**	2.46	96 h	**12.85**	8.12	**3.86**	1.47	**1.18**	1.08
7 d	**14.99**	2.95	**0.00**	0.00	**0.00**	0.00	7 d	**14.99**	2.95	**0.00**	0.00	**0.00**	0.00	7 d	**9.95**	3.29	**3.31**	0.80	**1.72**	1.84

**Table 2:** Distribution of regulatory T cell populations according to Foxp3 expression along time.

	CD4+CD25+Foxp3+					
			Low		High	
Time	Cells/ul ± SD	% of CD4	Cells (% of Foxp3+)	FI	Cells (% of Foxp3+)	FI
1h	77.8 ± 40.3	3.3	84.6	513.2 ± 27.7	15.4	1532.4 ± 182.5
2d	5.2 ± 2.4	1.5	70.1	594.4 ± 147.7	29.9	1442.2 ± 468.1
4d	4.3 ± 2.9	1.0	92.6	438 ± 89.6	7.4	434.8 ± 595.5
7d	1.6 ± 1.4	1.7	100.0	522.7 ± 92.5	0.0	0.0 ± 0.0
	**CD8+Foxp3+**					
			**Low**		**High**	
**Time**	**Cells/ul ± SD**	**% of CD8+**	**Cells (% of Foxp3+)**	**FI**	**Cells (% of foxp3+)**	**FI**
1h	43.7 ± 63.4	5.03	85.27	485.5 ± 31.5	14.73	1263.4 ± 776.7
2d	4.5 ± 5.0	2.20	74.38	346.0 ± 205.3	25.62	520.6 ± 761.2
4d	6.6 ± 8.6	2.42	78.26	373.0 ± 219.1	21.74	286.4 ± 640.4
7d	20.5 ± 17.6	33.0	98.13	404.0 ± 8.5	1.9	312.3 ± 541.0

## Data Availability

Not applicable.
